# Cardio-oncology in multidisciplinary synergy: addressing the rising burden of cancer therapy-related cardiovascular toxicity (CTR-CVT) in advancing oncology practices

**DOI:** 10.3389/or.2026.1852886

**Published:** 2026-06-12

**Authors:** Putri Dwi Astuti, Eko Adhi Pangarsa, Andreas Arie Setiawan, Daniel Rizky, Budi Setiawan, Charles Limantoro, Damai Santosa, Catharina Suharti

**Affiliations:** 1 Division of Hematology and Medical Oncology, Department of Internal Medicine, Faculty of Medicine, Diponegoro University, Semarang, Indonesia; 2 Division of Hematology and Medical Oncology, Department of Oncology, Dr. Kariadi General Hospital, Semarang, Indonesia; 3 Department of Cardiovascular, Dr. Kariadi General Hospital, Semarang, Indonesia; 4 Division of Cardiology, Department of Internal Medicine, Faculty of Medicine, Diponegoro University, Semarang, Indonesia

**Keywords:** cancer therapy, cardio-oncology, cardiovascular toxicity, multidisciplinary care, risk stratification

## Abstract

**Introduction:**

Improvements in cancer detection and the development of modern anticancer therapies have markedly increased survival rates among patients with malignancies, resulting in a rapidly expanding survivor population. Alongside these advances, cardiovascular complications related to cancer treatment have emerged as a major clinical concern, significantly affecting long-term morbidity and mortality. The growing intersection between oncology and cardiovascular medicine has led to the development of cardio-oncology, a multidisciplinary field focused on balancing effective cancer treatment with cardiovascular safety. This review discusses current perspectives on the mechanisms, risk assessment, prevention, surveillance, and management of cancer therapy–related cardiovascular toxicity (CTR-CVT) in contemporary oncology practice.

**Methods:**

A narrative literature review was conducted using electronic databases including PubMed, Scopus, and ScienceDirect. Relevant studies published between 2015 and 2025 were identified using predefined keywords related to cardio-oncology and cardiovascular toxicity. Eligible articles included clinical trials, observational studies, guidelines, and systematic reviews focusing on CTR-CVT. Studies were screened based on relevance, and key findings were synthesized narratively.

**Key-content and findings:**

CTR-CVT comprises diverse cardiovascular complications associated with both conventional and emerging anticancer therapies. Risk stratification tools such as HFA-ICOS facilitate early identification of high-risk patients and guide surveillance strategies. Early multidisciplinary management, particularly for severe toxicities such as immune checkpoint inhibitor–associated myocarditis, is essential to optimize both cardiovascular and oncologic outcomes.

**Conclusion:**

CTR-CVT represents a major challenge in contemporary oncology care. A multidisciplinary cardio-oncology approach integrating early risk assessment, preventive strategies, and structured monitoring is essential to balance oncologic efficacy with cardiovascular safety. Future research should focus on personalized risk prediction and targeted cardioprotection strategies.

## 1 Introduction

Over the past 3 decades, advances in cancer detection and treatment including chemotherapy, targeted therapy, and immunotherapy have significantly improved survival rates (1). Consequently, the population of cancer survivors continues to rise globally. However, this improvement has revealed a parallel and often underrecognized burden of cardiovascular disease (CVD) among cancer survivors (2, 3).

Cardiovascular disease has emerged as a leading cause of non-cancer-related morbidity and mortality in this population, with a prevalence that exceeds that of the general population (4). This phenomenon is driven by a combination of shared risk factors such as hypertension, diabetes, dyslipidemia, and aging and the direct cardiotoxic effects of anticancer therapies, collectively referred to as cancer therapy–related cardiovascular toxicity (CTR-CVT) (5). Beyond the cardiovascular complications induced by anticancer therapies, recent evidence has also highlighted the emerging concept of reverse cardio-oncology, in which cardiovascular disease itself may contribute to increased cancer incidence and progression through shared inflammatory, metabolic, and immune-related mechanisms. This bidirectional relationship further underscores the complexity and expanding scope of cardio-oncology research (4, 5).

CTR-CVT encompasses a wide range of clinical manifestations, including left ventricular dysfunction, coronary artery disease, arrhythmias, hypertension, thromboembolic events, myocarditis, and pericardial disease (Table 1). These complications are associated with various anticancer agents, including anthracyclines, HER2-targeted therapies, vascular endothelial growth factor (VEGF) inhibitors, tyrosine kinase inhibitors, immune checkpoint inhibitors, and thoracic radiotherapy (6,7).

**TABLE 1:** Summary of major anticancer therapies, associated cardiovascular toxicities, baseline cardiovascular assessment, and recommended monitoring strategies in cardio-oncology practice.

Anticancer agent/Class	Major cardiovascular toxicities	Baseline assessment	Recommended monitoring
Anthracyclines	Left ventricular dysfunction, heart failure, cardiomyopathy	ECG, troponin, NT-proBNP, TTE with LVEF and GLS	Serial TTE and biomarkers during therapy; closer surveillance in high-risk patients
HER2-targeted therapies	Reversible left ventricular dysfunction, heart failure	ECG, TTE with LVEF and GLS	Echocardiography every 3 months during therapy; continued post-treatment monitoring in selected high-risk patients
Fluoropyrimidines (5-FU, capecitabine)	Coronary vasospasm, ischemia, myocardial infarction, arrhythmias	Cardiovascular risk assessment, ECG, blood pressure, lipid and glucose profile	Clinical monitoring for ischemic symptoms and ECG monitoring during treatment
VEGF inhibitors	Hypertension, arterial thrombosis, heart failure, vascular dysfunction	Blood pressure assessment, ECG, TTE in selected patients	Frequent blood pressure monitoring, periodic ECG and cardiac evaluation
BCR-ABL tyrosine kinase inhibitors	Hypertension, arterial occlusive disease, QT prolongation, metabolic abnormalities	ECG, blood pressure, glucose and lipid profile, ankle–brachial index in selected cases	Periodic cardiovascular risk assessment and ECG monitoring
RAF/MEK inhibitors	Hypertension, cardiomyopathy, thromboembolism	ECG, TTE with LVEF and GLS, blood pressure assessment	Serial blood pressure and echocardiographic monitoring
Immune checkpoint inhibitors (ICIs)	Myocarditis, arrhythmias, heart failure, pericardial disease	ECG, troponin, NT-proBNP, TTE	Serial ECG and troponin monitoring, especially during early treatment cycles
CAR-T cell therapy	Cytokine release syndrome, heart failure, arrhythmias, hypotension	ECG, troponin, NT-proBNP, TTE	Continuous hemodynamic monitoring, biomarkers, and cardiac imaging as indicated
Tumor-infiltrating lymphocyte (TIL) therapy	Cytokine release syndrome, arrhythmias, myocardial dysfunction	ECG, cardiac biomarkers, TTE	Clinical and cardiac monitoring during therapy administration
CDK4/6 inhibitors	QT prolongation, arrhythmias	ECG, electrolyte assessment	Serial ECG and electrolyte monitoring during therapy
Thoracic radiotherapy	Coronary artery disease, valvular disease, pericardial disease, fibrosis	Cardiovascular risk assessment, baseline TTE	Long-term surveillance for delayed cardiovascular complications

In response to this growing challenge, cardio-oncology has emerged as a multidisciplinary field that integrates cardiology and oncology to optimize patient outcomes (8). This approach emphasizes pre-treatment cardiovascular risk assessment, individualized monitoring strategies, early detection of toxicity, and timely intervention.

Unlike prior guideline-focused reviews primarily centered on established cardiotoxicity surveillance frameworks, this review integrates contemporary evidence on emerging anticancer therapies, individualized risk-adapted monitoring strategies, and multidisciplinary implementation models in cardio-oncology practice. Furthermore, this review emphasizes practical challenges in integrating cardio-oncology services across diverse healthcare settings, including resource-limited environments, while also discussing future perspectives such as artificial intelligence–driven risk prediction and precision cardio-oncology approaches.

## 2 Methods

### 2.1 Study design

The literature search across PubMed/MEDLINE, Scopus, and ScienceDirect initially identified approximately 462 articles published between 2015 and 2025. After removal of duplicates and screening of titles and abstracts for relevance to cardio-oncology and CTR-CVT, 118 articles underwent full-text evaluation. Following assessment for relevance, methodological quality, and clinical applicability, 36 articles were ultimately included in the narrative synthesis. The selected literature consisted of international guidelines, clinical trials, observational studies, systematic reviews, and meta-analyses addressing mechanisms, risk stratification, prevention, surveillance, and management of CTR-CVT in contemporary oncology practice.

### 2.2 Search strategy

The search strategy employed a combination of keywords and Medical Subject Headings (MeSH) related to the topic, including “cardio-oncology”, “cardiotoxicity”, “cancer therapy-related cardiovascular toxicity”, “anthracycline cardiomyopathy”, “HER2 cardiotoxicity”, “immune checkpoint inhibitor myocarditis”, and “cardiovascular complications of cancer therapy”. Boolean operators (AND, OR) were applied to refine and optimize the search results.

### 2.3 Eligibility criteria

Studies were considered eligible if they discussed cardiovascular toxicity associated with cancer therapy, involved adult human populations, were published in peer-reviewed journals, and were written in English. Eligible study designs included clinical trials, observational studies, systematic reviews, meta-analyses, and international clinical guidelines. Studies were excluded if they were case reports with limited generalizability, focused exclusively on pediatric populations, or were not published in English.

### 2.4 Study selection

All identified records were initially screened by title and abstract to assess relevance. Full-text articles of potentially eligible studies were subsequently reviewed to confirm inclusion. Additional relevant studies were identified through manual searching of reference lists from selected articles to ensure comprehensive coverage of the literature.

### 2.5 Data extraction and synthesis

Data extraction was performed by collecting key information from each included study, including study design, population characteristics, type of anticancer therapy, cardiovascular outcomes, and principal findings. Given the heterogeneity in study designs and reported outcomes, the findings were synthesized using a qualitative narrative approach rather than quantitative meta-analysis.

### 2.6 Quality appraisal

The methodological quality of the included studies was assessed using general appraisal principles based on study design, hierarchy of evidence, consistency of findings, and clinical relevance to cardio-oncology practice. Formal scoring systems were not applied, in line with standard narrative review methodology.

## 3 Main analysis

**FIGURE 1:**
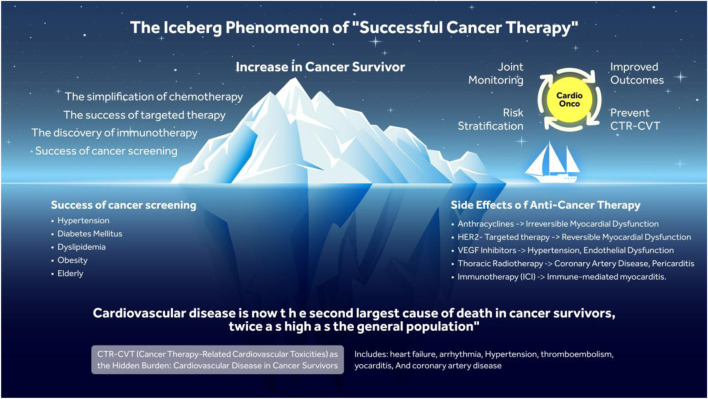
The hidden burden of CTR-CVT: cardiovascular disease in cancer survivors. Illustration of shared comorbidities and treatment-related toxicities contributing to cardiovascular disease among cancer survivors, often concealed beneath the apparent success of cancer therapy.

This narrative review identified and synthesized current evidence regarding the spectrum, mechanisms, risk stratification, prevention, monitoring, and management of CTR-CVT. The findings demonstrate that CTR-CVT represents a heterogeneous group of cardiovascular complications arising from both conventional and novel anticancer therapies. Figure 1 summarizes the major categories of CTR-CVT and illustrates the distinct cardiovascular manifestations associated with various anticancer therapies, highlighting the heterogeneity of cardiotoxic mechanisms.

CTR-CVT encompasses a wide range of clinical manifestations, including left ventricular dysfunction, heart failure, coronary artery disease, arrhythmias, hypertension, thromboembolic events, myocarditis, and pericardial disease. These complications are associated with multiple classes of anticancer agents, each characterized by distinct pathophysiological mechanisms. Anthracyclines remain the most well-established cause of irreversible cardiotoxicity through oxidative stress and topoisomerase IIβ inhibition, leading to cardiomyocyte apoptosis and dose-dependent cardiomyopathy (9). In contrast, HER2-targeted therapies induce predominantly reversible myocardial dysfunction by disrupting cardiomyocyte survival pathways (10).

Other therapies demonstrate unique toxicity profiles. Fluoropyrimidines such as 5-fluorouracil are primarily associated with coronary vasospasm, while VEGF inhibitors and tyrosine kinase inhibitors commonly induce hypertension and vascular dysfunction. Immune checkpoint inhibitors (ICIs) introduce a distinct immune-mediated cardiotoxicity, particularly myocarditis, which can be severe and potentially fatal (11). Additionally, radiotherapy contributes to long-term cardiovascular complications through endothelial injury, fibrosis, and accelerated atherosclerosis (12).

### 3.1 Risk stratification and pre-treatment cardiovascular assessment

**FIGURE 2:**
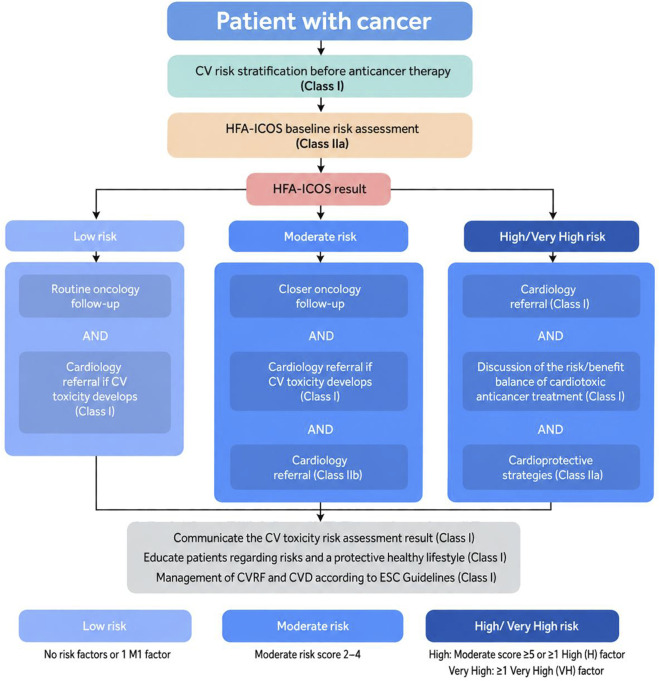
Cardio-oncology approach to patients based on risk stratisfication. Adapted from 2022 ESC Guidelines on Cardio-oncology. a. Cardio-oncology referral is recommended when available; alternatively, patients should be referred to a specialized cardiologist with expertise in managing CVD in patients with cancer.

Risk stratification represents a fundamental component of cardio-oncology care, as it guides surveillance intensity, preventive strategies, and therapeutic decision-making throughout cancer treatment. The HFA-ICOS risk assessment tool, recommended by the 2022 European Society of Cardiology (ESC) guidelines, integrates multiple parameters including clinical history, pre-existing cardiovascular risk factors, imaging findings, biomarkers, and the cardiotoxic profile of anticancer therapies to classify patients into low-, moderate-, high-, and very high-risk categories (13). Figure 2 illustrates the HFA-ICOS framework and its role in supporting individualized cardiovascular surveillance and prevention strategies.

Comprehensive baseline cardiovascular evaluation should be performed before initiation of potentially cardiotoxic therapies. This assessment includes detailed history taking and physical examination, with emphasis on hypertension, diabetes mellitus, dyslipidemia, smoking history, prior cardiovascular disease, and other comorbidities that may increase susceptibility to CTR-CVT (19). Patients identified as having substantial cardiovascular risk may require additional cardiology consultation before anticancer therapy is initiated (20).

Electrocardiography and cardiac biomarkers are important components of baseline assessment. A 12-lead electrocardiogram (ECG) may detect conduction abnormalities or QTc prolongation, while biomarkers such as troponin and NT-proBNP provide additional information regarding myocardial injury and baseline cardiac stress (21).

Cardiac imaging remains central to pre-treatment risk evaluation. Transthoracic echocardiography (TTE) is the preferred first-line modality because of its accessibility and ability to evaluate left ventricular ejection fraction (LVEF) and global longitudinal strain (GLS), both of which are essential for early detection of subclinical dysfunction (14). In patients with suboptimal echocardiographic windows or when greater tissue characterization is required, cardiac magnetic resonance (CMR) may be used as a second-line modality and is considered the reference standard for cardiac functional assessment (22).

Alternative imaging modalities, including multigated acquisition (MUGA) scanning, may be considered when echocardiography or CMR is unavailable, although concerns about radiation exposure limit routine use. Nuclear imaging techniques such as SPECT and PET may additionally provide insights into myocardial perfusion and metabolic alterations associated with cardiotoxic therapies, particularly anthracyclines, although their use remains selective in clinical practice (23,24).

Following baseline evaluation, monitoring strategies should be individualized according to the patient’s baseline cardiovascular risk and the specific anticancer agent administered. High-risk patients generally require more intensive surveillance protocols and may benefit from preventive interventions including optimization of cardiovascular risk factors, lifestyle modification, and cardioprotective therapies such as ACE inhibitors, beta-blockers, statins, or dexrazoxane in selected populations (15, 16).

Management of established CTR-CVT depends on the type and severity of toxicity and often requires multidisciplinary collaboration between oncologists and cardiologists. Certain toxicities, such as immune checkpoint inhibitor (ICI)-associated myocarditis, require prompt immunosuppressive therapy, whereas cytokine release syndrome related to CAR-T therapy may necessitate targeted anti–IL-6 treatment (17, 18). Overall, these approaches highlight the importance of individualized and risk-adapted cardio-oncology care throughout the continuum of cancer treatment.

### 3.2 Prevention of CTR-CVT

#### 3.2.1 General preventive strategies

Prevention of CTR-CVT begins at the time of cancer diagnosis and is guided by cardiovascular risk stratification. Preventive strategies aim to minimize cardiovascular toxicity while maintaining optimal oncologic outcomes. These strategies include optimization of traditional cardiovascular risk factors, lifestyle interventions, and cardioprotective pharmacotherapy (25).

Optimization of cardiovascular health is guided by the American Heart Association’s Life’s Simple 7, which includes blood pressure control, maintenance of a healthy body mass index, smoking cessation, glycemic control, adherence to a heart-healthy diet, regular physical activity, and cholesterol management (25, 26).

Pharmacologic cardioprotection is recommended for high- and very high-risk patients. Current therapeutic options include ACE inhibitors or ARBs, beta-blockers, statins, dexrazoxane, and liposomal anthracyclines. ESC 2022 guidelines provide a Class IIa recommendation for the use of these agents in selected high-risk populations to mitigate declines in cardiac function during therapy (26).

#### 3.2.2 Agent-specific preventive strategies

Anthracyclines are associated with a high risk of cardiotoxicity, particularly in patients with reduced baseline left ventricular ejection fraction (LVEF); therefore, all patients should undergo baseline transthoracic echocardiography (TTE) with assessment of LVEF and global longitudinal strain (GLS), while cardiac biomarkers are recommended in high-risk individuals, and dexrazoxane or liposomal anthracyclines should be considered when cumulative doses exceed 300 mg/m^2^. HER2-targeted agents are associated with left ventricular dysfunction in approximately 15%–20% of patients, necessitating baseline and serial monitoring of cardiac function using TTE, with biomarkers considered in selected high-risk cases despite their lower sensitivity (27).

Fluoropyrimidines, such as 5-fluorouracil, can induce coronary vasospasm leading to angina or myocardial infarction; thus, baseline evaluation should include cardiovascular risk assessment, ECG, blood pressure, lipid profile, and glycemic status, with TTE recommended in symptomatic patients (28). VEGF inhibitors are most commonly associated with hypertension, making baseline and serial blood pressure monitoring essential, along with ECG evaluation, and strict blood pressure control should be ensured before and during therapy. RAF/MEK inhibitors are linked to hypertension, thromboembolic events, and cardiomyopathy, requiring baseline assessment of LVEF, GLS, and ECG, as well as optimal blood pressure control prior to treatment initiation (29).

Cardiovascular toxicity associated with BCR-ABL tyrosine kinase inhibitors varies by agent, with baseline evaluation including ECG, blood pressure, glucose, and lipid profile, and additional assessments such as ankle–brachial index and echocardiography recommended for selected patients. Finally, immune checkpoint inhibitor (ICI) therapy can lead to immune-mediated cardiotoxicity, particularly myocarditis; therefore, comprehensive baseline evaluation with ECG, cardiac biomarkers, and TTE is recommended for all patients (28, 29).

### 3.3 Monitoring during cancer therapy

#### 3.3.1 General principles of surveillance

Continuous cardiovascular monitoring during anticancer therapy is essential for early detection of CTR-CVT. Surveillance strategies should be individualized based on baseline risk and the specific anticancer agent. Monitoring includes clinical evaluation, cardiac biomarkers, and imaging modalities. The frequency and intensity of monitoring are determined by risk stratification using tools such as HFA-ICOS and by the cardiotoxic profile of the therapy. Early detection of subclinical toxicity allows for timely intervention and improved outcomes (30).

#### 3.3.2 Agent-specific monitoring strategies

Anthracycline therapy requires serial evaluation using TTE and cardiac biomarkers, with increased frequency in high-risk patients. HER2-targeted therapy requires echocardiographic assessment every 3 months during treatment. VEGF inhibitors necessitate close blood pressure monitoring, particularly during early treatment cycles (31). HER2-targeted therapy generally requires echocardiographic surveillance every 3 months during treatment, with continued post-treatment monitoring recommended in high-risk patients or those who develop cardiac dysfunction, typically extending for at least 6–12 months after therapy completion depending on clinical status and recovery of cardiac function (31, 32).

For BCR-ABL TKIs, monitoring includes periodic assessment of cardiovascular risk factors and targeted imaging depending on the specific agent used. RAF/MEK inhibitors require serial blood pressure monitoring and echocardiographic evaluation in high-risk individuals. ICI therapy requires frequent monitoring of ECG and troponin levels, particularly during treatment cycles, to detect myocarditis early. Chimeric antigen receptor T-cell therapy (CAR-T) and tumor-infiltrating lymphocyte (TIL) therapies require baseline and ongoing assessment using ECG, biomarkers, and imaging due to the risk of cytokine release syndrome (31,32).

### 3.4 Management of cardiovascular toxicity

Management of CTR-CVT requires a multidisciplinary approach involving both cardiologists and oncologists, with the primary goal of minimizing cardiovascular complications while maintaining the efficacy of anticancer therapy; therefore, management strategies should be individualized based on the specific anticancer agent and clinical presentation. In anthracycline-related cardiotoxicity, management is guided by symptom severity, where mild asymptomatic cases may allow continuation of therapy with close monitoring and initiation of heart failure treatment, while moderate to severe cases require treatment interruption and guideline-directed heart failure therapy, and severe symptomatic cases necessitate permanent discontinuation of anthracyclines (33).

A similar approach applies to HER2-related cardiotoxicity, in which therapy may be continued in mild cases but should be interrupted in more severe dysfunction, with prompt initiation of heart failure therapy and reassessment guiding reintroduction of treatment. Immune checkpoint inhibitor (ICI) myocarditis requires urgent recognition and management, including early initiation of high-dose intravenous corticosteroids followed by gradual tapering, with escalation to additional immunosuppressive therapies in refractory cases. Cardiovascular complications associated with CAR-T and tumor-infiltrating lymphocyte (TIL) therapies, particularly those related to cytokine release syndrome, are managed with anti–IL-6 therapy, such as tocilizumab, in combination with corticosteroids and supportive cardiac care.

QT interval prolongation associated with CDK4/6 inhibitors requires discontinuation of offending agents in severe cases, correction of electrolyte imbalances, and close ECG monitoring, with therapy withheld when QTc exceeds 500 ms. Finally, Takotsubo syndrome, which may be triggered by malignancy or anticancer therapy, necessitates temporary interruption of cancer treatment and supportive management, with diagnosis established through multimodal imaging and exclusion of obstructive coronary artery disease (34).

Immune checkpoint inhibitor myocarditis requires urgent recognition due to its high mortality risk. Initial management generally involves prompt initiation of high-dose corticosteroid therapy, followed by gradual tapering based on clinical response and biomarker improvement. Elevated troponin levels, ventricular arrhythmias, and reduced left ventricular function are associated with poorer prognosis. Permanent discontinuation of immune checkpoint inhibitor therapy is generally recommended in severe or life-threatening myocarditis cases (33, 34). Several clinical and laboratory parameters have been associated with poorer prognosis in ICI myocarditis, including markedly elevated troponin levels, reduced left ventricular ejection fraction, ventricular arrhythmias, conduction abnormalities, and hemodynamic instability. Early identification of these high-risk features is important to guide monitoring intensity and therapeutic escalation. The decision to resume or permanently discontinue ICI therapy should be individualized according to the severity of toxicity and clinical recovery. Mild cases with complete resolution may allow cautious reintroduction under close surveillance, whereas severe or life-threatening myocarditis, persistent ventricular dysfunction, significant arrhythmias, or recurrent toxicity generally warrant permanent discontinuation of immunotherapy (32–34).

### 3.5 Consensus and multidisciplinary cardio-oncology care

**FIGURE 3:**
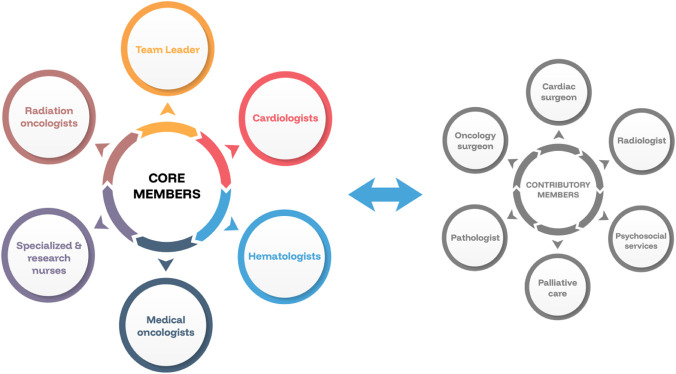
Structure of a cardio-oncology multi disciplinary team, consisisting of core members and contributory members collaborating in patient-centered care.

The ESMO consensus outlines four key pillars in the management of cardiovascular disease in patients undergoing cancer therapy, namely, pre-treatment risk stratification, primary prevention, surveillance during therapy, and early management of toxicity, all of which require a coordinated multidisciplinary approach to balance oncologic benefit with cardiovascular safety. In this context, the implementation of cardio-oncology programs relies on a structured multidisciplinary team (MDT) involving cardiologists, oncologists, and other allied healthcare professionals, where clinical decision-making is individualized and guided through collaborative discussions (35). The establishment of dedicated cardio-oncology units (COUs) further enhances coordination of care by integrating multiple specialties within a unified framework, thereby improving patient outcomes. Care delivery follows a continuum that spans pre-treatment cardiovascular assessment, ongoing monitoring during therapy, and long-term post-treatment follow-up, ensuring early detection and management of both acute and late cardiovascular complications. Importantly, in resource-limited settings, the implementation of such programs requires pragmatic adaptation, emphasizing cost-effective strategies, simplified monitoring protocols, and efficient referral systems without compromising the quality of care (36). Figure 3 demonstrates the multidisciplinary workflow of cardio-oncology care, emphasizing coordination between oncology, cardiology, and supportive care teams throughout the continuum of cancer treatment.

In resource-limited settings, cardio-oncology programs may adopt pragmatic strategies such as prioritizing focused cardiovascular risk assessment, utilizing echocardiography selectively for high-risk patients, implementing simplified biomarker-based surveillance protocols, and strengthening referral collaboration between oncology and cardiology services. For example, periodic troponin assessment combined with symptom-based evaluation may provide a more feasible surveillance approach in centers with limited imaging availability.

### 3.6 Future directions

Future research in cardio-oncology should focus on the development of precision-based risk prediction models integrating imaging, biomarkers, genomics, and artificial intelligence algorithms to enable individualized prevention strategies. Biomarker-guided phenotyping may further improve early identification of patients at highest risk for CTR-CVT. Additionally, the growing population of cancer survivors highlights the need for long-term survivorship models integrating cardiovascular monitoring into routine oncology follow-up. Global disparities in access to cardio-oncology services also remain an important challenge, particularly in low- and middle-income countries, where simplified and cost-effective implementation strategies are required.

## 4 Conclusion

Cardio-oncology has emerged as a critical discipline in modern cancer care, addressing the growing burden of CTR-CVT in an era of improving cancer survival. This review highlights that CTR-CVT encompasses a broad and complex spectrum of cardiovascular complications, with distinct mechanisms and clinical manifestations across different anticancer therapies.

Effective management requires a comprehensive and individualized approach that begins with pre-treatment cardiovascular risk stratification, followed by tailored preventive strategies and structured surveillance throughout the course of therapy.
